# Overexpression of *OsHMA3* enhances Cd tolerance and expression of Zn transporter genes in rice

**DOI:** 10.1093/jxb/eru340

**Published:** 2014-08-23

**Authors:** Akimasa Sasaki, Naoki Yamaji, Jian Feng Ma

**Affiliations:** Institute of Plant Science and Resources, Okayama University, Chuo 2-20-1, Kurashiki, Japan

**Keywords:** OsHMA3, Cd, overexpression, *Oryza sativa*, Zn transporter, tolerance.

## Abstract

As a member of the heavy metal ATPase (HMA) family, OsHMA3 is a tonoplast-localized transporter for Cd in the roots of rice (*Oryza sativa*). Overexpression of *OsHMA3* selectively reduces Cd accumulation in the grain. Further characterization in the present study revealed that overexpression of *OsHMA3* also enhances the tolerance to toxic Cd. The growth of both the roots and shoots was similar in the absence of Cd between an *OsHMA3*-overexpressed line and vector control, but the Cd-inhibited growth was significantly alleviated in the *OsHMA3*-overexpressed line. The overexpressed line showed higher Cd concentration in the roots, but lower Cd concentration in the shoots compared with the wild-type rice and vector control line, indicating that overexpression of *OsHMA3* enhanced vacuolar sequestration of Cd in the roots. The Zn concentration in the roots of the *OsHMA3*-overexpressed line was constantly higher than that of vector control, but the Zn concentration in the shoots was similar between the overexpressed line and vector control. Five transporter genes belonging to the ZIP family were constitutively up-regulated in the *OsHMA3*-overexpressed line. These results suggest that shoot Zn level was maintained by up-regulating these genes involved in the Zn uptake/translocation. Taken together, overexpression of *OsHMA3* is an efficient way to reduce Cd accumulation in the grain and to enhance Cd tolerance in rice.

## Summary

Overexpression of a tonoplast-localized transporter, OsHMA3, enhanced Cd tolerance and selectively reduced Cd accumulation in the shoots, but shoot Zn level was maintained by up-regulating genes involved in Zn uptake/translocation.

## Introduction

Rice (*Oryza sativa*) as a staple food is a major source of dietary intake of cadmium (Cd), which accounts for more than 40% of total Cd intake in Japan (Shimbo *et al.*, 2001). Itai-itai disease, which is mainly due to kidney tubule damage, was caused in the past by taking rice with high Cd (Horiguchi *et al.*, 1994). Therefore, control of Cd accumulation in rice grain is a very important issue of food safety, especially in areas with high Cd contamination.

Recently great progress has been made in understanding molecular mechanisms of Cd accumulation in rice (Clemens *et al.*, 2013). The uptake of Cd in the roots is mediated by OsNramp5, a plasma-membrane-localized transporter belonging to the Nramp family (Sasaki *et al.*, 2012). *OsNramp5* is expressed in the roots through the whole growth period, and is polarly localized at the distal side of the exodermis and endodermis (Sasaki *et al.*, 2012). Knockout of *OsNramp5* resulted in decreased Cd uptake and Cd accumulation in the grain (Sasaki *et al.*, 2012; Ishikawa *et al.*, 2012). However, as OsNramp5 is also a major transporter responsible for Mn uptake, knockout of this gene caused reduced growth and yield owing to Mn deficiency (Sasaki *et al.*, 2012), although this effect was not observed in another study (Ishikawa *et al.*, 2012). In addition to OsNramp5, OsIRT1 and OsIRT2, belonging to the ZIP transporters, and OsNramp1 have also been proposed to contribute to Cd uptake, but their actual contribution is unknown (Clemens *et al.*, 2013).

Cd taken up into the cells is sequestered into the vacuoles by OsHMA3, a member of HMA family (Ueno *et al.*, 2010; Miyadate *et al.*, 2011). OsHMA3 is localized to the tonoplast of all root cells (Ueno *et al.*, 2010). Mutation of OsHMA3 through single amino acid substitution resulted in high Cd accumulation in the grain in some high Cd-accumulating rice cultivars such as Anjana Dhan and Jarjan (Ueno *et al.*, 2010; Miyadate *et al.*, 2011; Ueno *et al.*, 2011). This allelic variation in OsHMA3 accounts for a major QTL for Cd accumulation detected on chromosome 7 (Ueno *et al.*, 2009). On the other hand, overexpression of *OsHMA3* reduced Cd accumulation in the grain, but did not affect the concentration of Fe and Zn (Ueno *et al.*, 2010).

The root-to-shoot translocation of Cd was mediated by OsHMA2, a homologue of OsHMA3 (Satoh-Nagasawa *et al.*, 2012; Takahashi *et al.*, 2012; Yamaji *et al.*, 2013). However, differently from OsHMA3, OsHMA2 is localized at the plasma-membrane of root pericycle cells (Yamaji *et al.*, 2013). Knockout of *OsHMA2* resulted in marked reduction of Cd in the shoots and grain (Satoh-Nagasawa *et al.*, 2012; Takahashi *et al.*, 2012; Yamaji *et al.*, 2013), but also caused reduction of the growth and grain yield (Yamai *et al.*, 2013). This is because OsHMA2 is also a Zn transporter, which is responsible for preferential distribution of Zn to the developing tissues. At the vegetative and reproductive growth stages, OsHMA2 is also localized at the phloem region of the nodes, which is involved in the inter-vascular transfer of Zn and Cd (Yamaji *et al.*, 2013). Another transporter, OsLCT1, also mediates xylem-to-phloem transfer of Cd (but not Zn) in node I of rice (Uraguchi *et al.*, 2011). *OsLCT1* was expressed in both enlarged vascular bundles and diffuse vascular bundles of the node. Knockdown of *OsLCT1* also resulted in decreased Cd in the phloem sap and Cd accumulation in the grain (Uraguchi *et al.*, 2011).

Identification of transporters involved in Cd accumulation as described above indicates that Cd is transported through transporters for essential metals such as Zn and Mn. Therefore, manipulation of these transporters will also affect the uptake and distribution of these essential metals, causing growth inhibition. However, overexpression of *OsHMA3* only affected Cd accumulation in the grain, but not Fe and Zn (Ueno *et al.*, 2010). To understand the underlying mechanisms, in the present study we further characterized an *OsHMA3*-overexpressed line. We found that overexpression of *OsHMA3* enhanced the tolerance to Cd toxicity. Furthermore, we found that although OsHMA3 is also responsible for vacuolar sequestration of Zn, the Zn level in the shoot of *OsHMA3*-overexpressed line is maintained by up-regulating five ZIP genes implicated in Zn uptake and translocation.

## Materials and methods

### Plant materials and growth conditions

Seeds of wild-type rice (WT, cv. Nipponbare), an empty vector line and an *OsHMA3* overexpressed (OX) line prepared before (Ueno *et al.*, 2010), were germinated in the dark at 30 °C for 2 d. The germinated seeds were transferred on a net floating on a 0.5mM CaCl_2_ solution in a 1.5 l plastic container. The solution was changed once every 2 d. After growth for 7–10 d at 25 °C, seedlings with similar size were transferred into a 3-l pot containing half-strength Kimura B solution (Zheng *et al.*, 2012). The nutrient solution was changed once every 2 d. The plants were grown in a closed green house with natural light at 25 °C. All experiments were repeated at least once with 3–4 biological replicates for each.

### Evaluation of Cd tolerance

Seedlings (24-d-old) were exposed to one-half strength Kimura B solution containing 0, 100, and 1000nM Cd in a 1.2-L pot. The solution was renewed every 2 d. After 22 d, the roots were washed with 5mM CaCl_2_ for three times and separated from the shoots. The samples were dried in an oven at 70 °C for at least 3 d. After the dry weight of the roots and shoots was recorded, the samples were subjected to digest for mineral analysis as described below. Three biological replicates (one plant for each) were made for each treatment.

### Kinetic study of Cd and Zn uptake

To investigate time-dependent uptake of Cd, the seedlings (38-day-old) were exposed to a one-half-strength Kimura B solution containing 500nM Cd in a 1.2-l pot. The roots were sampled at 1, 3, 6, 12, and 24h after the exposure with four biological replicates (one plant for each). For investigation of Zn uptake, seedlings (27-day-old) were exposed to a half-strength Kimura B solution containing 500nM ^67^ZnCl_2_ with four biological replicates (one plant for each). The ^67^ZnCl_2_ stable isotope was purchased from Taiyo Nippon Sanso Corporation (Tokyo, Japan). At 0.5, 1, 3, 6, 12, and 24h, the roots were sampled.

The roots were washed in a 5mM CaCl_2_ solution for three times before harvest and then immediately frozen in liquid nitrogen. The samples were stored at –80 °C until use. Root cell sap was extracted and then subjected to Cd and Zn determination as described below.

### Uptake of other divalent metals

To investigate the effect of overexpression of *OsHMA3* on the uptake of other divalent metals, seedlings (28-d-old) of both OX and WT were exposed to Pb, Co, and Ni at 500nM in a nutrient solution without Zn with four biological replicates (one plant for each). After exposure for 24h, the roots were washed with 5mM CaCl_2_ solution for three times and separated from the shoots with a razor. After the samples were dried in an oven for at least 2 d, they were subjected to metal analysis as described below.

### Root cell sap extraction, sample digest, and mineral determination

The frozen samples were placed in ultra free-MC centrifugal filter units (0.2 µm, Millipore) at room temperature. After thawing for a short-time, the tubes were centrifuged at 20 400*g* for 10min to obtain the root cell sap.

The dried root and shoot samples were digested with HNO_3_ as described before (Zheng *et al.*, 2012).

The concentration of Zn, Fe, Mn, Cu, Cd, Ni, Pb, and Co in the cell sap and digest solution was determined by ICP-MS (Agilent 7700). The concentration of ^67^Zn was determined with an isotope mode.

### Expression analysis of ZIP genes

To compare expression of genes related to Zn transport, samples of both *OsHMA3*-overexpressed line and vector control were taken from the roots of 28-day-old seedlings exposed to 0 or 200nM Cd for 24h with three biological replicates. Total RNA was extracted by using an RNeasy Plant Mini Kit (Qiagen), which was converted to cDNA followed by DNase I (Invitrogen) treatment using the protocol supplied by the manufacturers of SuperScript II (Invitrogen). The cDNAs were amplified by SsoFast EvaGreen Supermix (Bio-Rad). The expression of ten ZIP genes was determined by quantitative real-time PCR using the primers listed in Supplementary Table S1 on CFX384 (Bio-Rad). The expression data were normalized by *Histone H3* and *Actin* as internal standards, and relative expression was calculated by the comparative cycle threshold method using CFX Manager software (Bio-Rad).

## Results

### Overexpression of OsHMA3 enhanced Cd tolerance

Two independent *OsHMA3*-overexpressed lines were used to evaluate Cd accumulation in brown rice (Ueno *et al.*, 2010). Both lines showed similar expression level of *OsHMA3* and phenotype (Cd accumulation). In the present study, one line was selected to further investigate the effect of overexpression of *OsHMA3* on Cd tolerance and other traits.

**Fig. 1.**
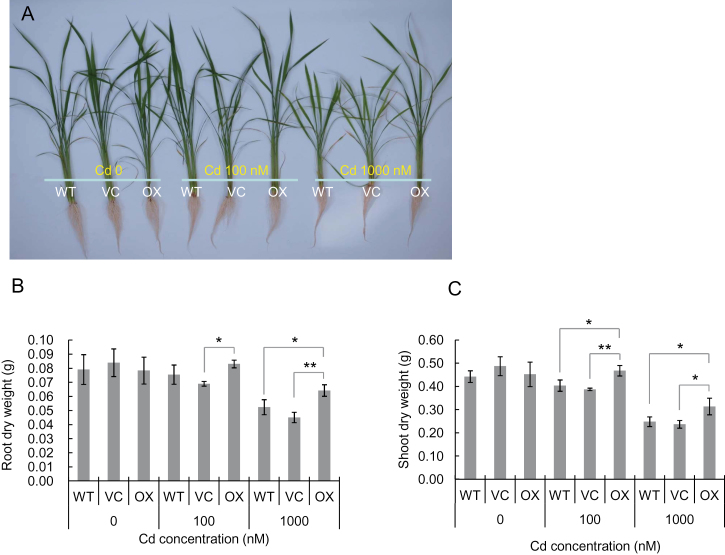
Effect of overexpression of *OsHMA3* on Cd tolerance in rice. (A) Phenotype of *OsHMA3* overexpressed line (OX), vector control line (VC), and non-transgenic wild-type rice (WT, cv. Nipponbare). (B) Root dry weight of the three lines. (C) Shoot dry weight of the three lines. All lines were cultivated in one-half strength Kimura B solution containing 0, 100, and 1000nM Cd for 22 d. Data are means±SD of three biological replicates. Statistical comparison was performed by one-way ANOVA followed by the Tukey’s test. All data were compared with the wild type, vector control, and overexpression line in each treatment (*P<0.05 and **P<0.01).

In the absence of Cd, similar growth was observed among wild-type rice (WT), vector control (VC), and the overexpressed line (OX) (Fig. 1A–C). At 100nM Cd, although the growth of the shoots of OX was slightly better than that of WT and VC, there was no significant difference among the three lines. However, at 1000nM Cd, the leaves of WT and VC showed severe chlorosis (Fig. 1A) and the dry weight of the shoots and roots were lower than OX (Fig. 1B, C). There was no difference in the growth between WT and VC at either Cd concentration, indicating that transformation did not affect the growth itself.

**Fig. 2.**
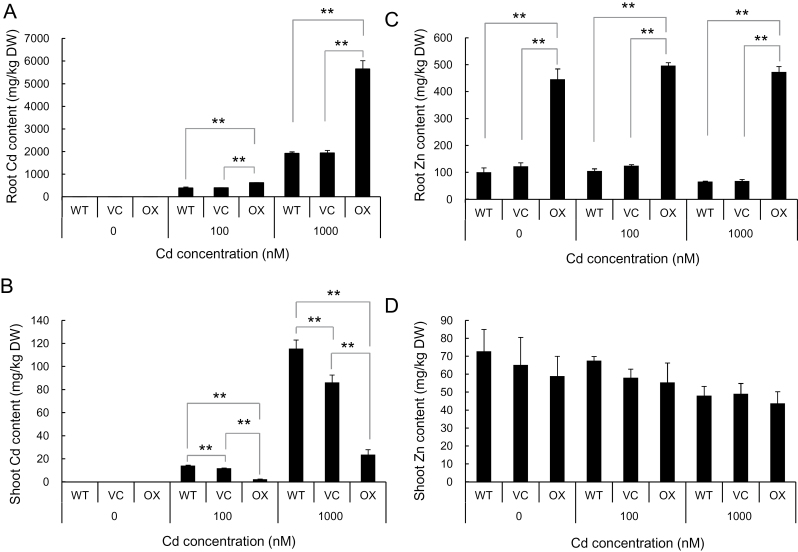
Concentration of Cd and Zn in the roots and shoots of the *OsHMA3* overexpressed line. An *OsHMA3* overexpressed line (OX), vector control line (VC), and non-transgenic wild-type rice (WT, cv. Nipponbare) were grown in one-half strength Kimura B solution containing 0, 100, and 1000nM Cd for 22 d. The concentration of Cd (A, B) and Zn (C, D) in the roots (A, C) and shoots (B, D) was determined with ICP-MS. Data are means±SD of three biological replicates. Statistical comparison was performed by one-way ANOVA followed by the Tukey’s test. All data were compared with the wild type, vector control and overexpression line in each treatment (*P<0.05 and **P<0.01).

**Fig. 3.**
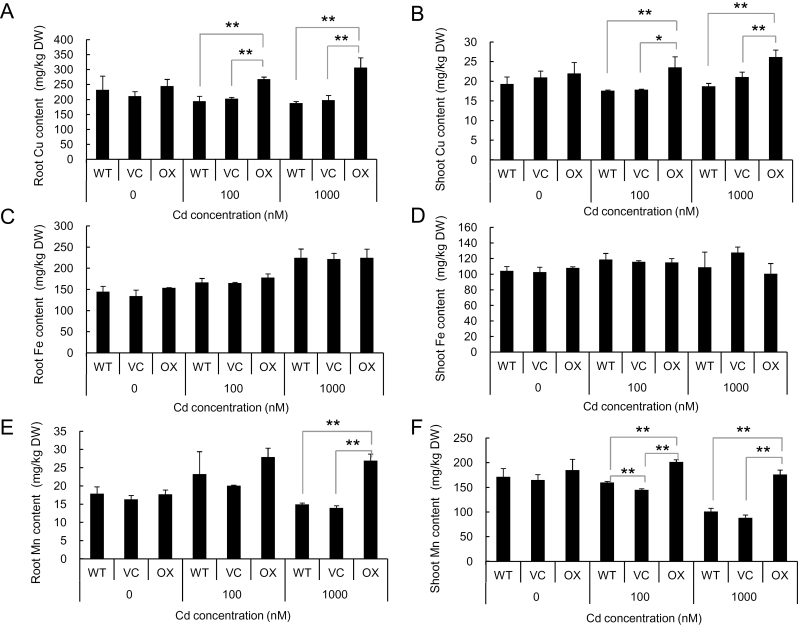
Concentration of Cu, Fe, and Mn in the roots and shoots. An *OsHMA3* overexpressed line (OX), vector control line (VC), and non-transgenic wild-type rice (WT, cv. Nipponbare) were grown in one-half strength Kimura B solution containing 0, 100, and 1000nM Cd for 22 d. The concentration of Cu (A, B), Fe (C, D), and Mn (E, F) in the roots (A, C, E) and shoots (B, D, F) was determined with ICP-MS. Data are means±SD of three biological replicates. Statistical comparison was performed by one-way ANOVA followed by the Tukey’s test. All data were compared with the wild type, vector control and overexpression line in each treatment (*P<0.05 and **P<0.01).

Mineral analysis showed that the Cd concentration in the roots was much higher in the OX than in the WT and VC at both lower and higher Cd concentrations (Fig. 2A). However, the Cd concentration in the shoots was significantly lower in the OX than in the WT and VC (Fig. 2B). Surprisingly, the Zn concentration in the roots of OX was always much higher than that of WT and VC (Fig. 2C), but the Zn concentration in the shoots was similar among the three lines under the same treatment, although high Cd (1000nM Cd) slightly decreased Zn concentration in all lines (Fig. 2D). There was no significant difference in the concentration of Cu, Fe, and Mn in the shoots and roots among the three lines in the absence of Cd (Fig. 3). However, in the presence of Cd, the concentration of Cu and Mn of both the roots and shoots was lower in the WT and VC than in the OX (Fig. 3A, B, E, F), but there was no difference in the Fe concentration of the roots and shoots among the three lines (Fig. 3C, D).

### Kinetics of Cd and Zn uptake

**Fig. 4.**
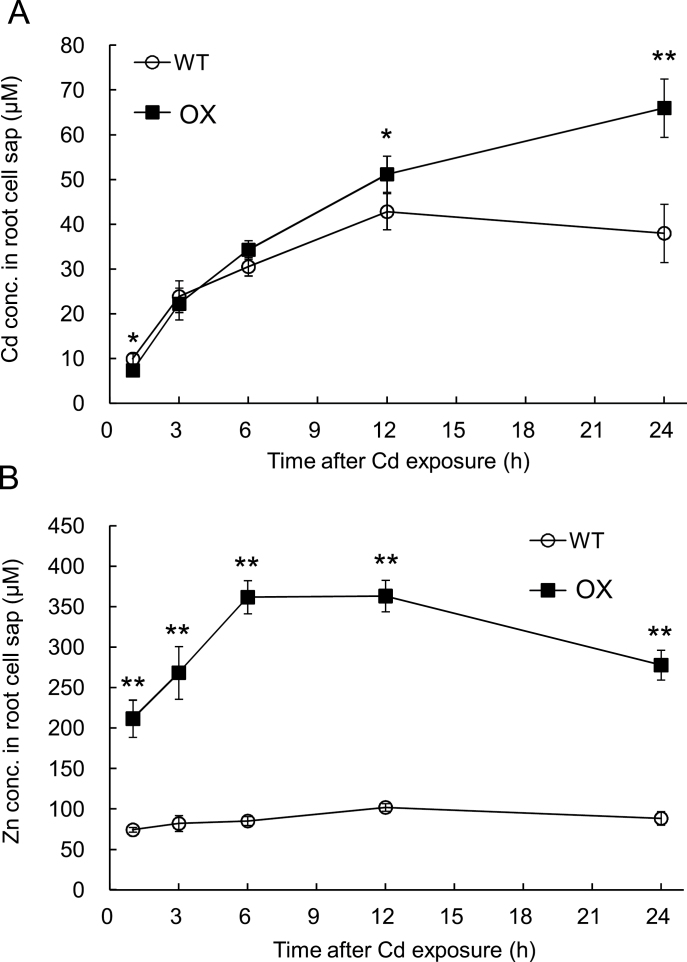
Time-dependent change of Cd and Zn in the root cell saps. Seedlings of an *OsHMA3* overexpressed line (OX) and non-transgenic wild-type rice (WT, cv. Nipponbare) were exposed to 500nM Cd for different times. Concentration of Cd (A) and Zn (B) in the root cell sap was determined with ICP-MS. Data are means±SD of four biological replicates. Asterisks indicate significant difference from WT at *P<0.05 and **P<0.01 by Student’s t-test.

To further investigate the effect of *OsHMA3* overexpression on the Cd uptake, a time-dependent change of Cd concentration in the root cell sap was monitored. Root cell sap mainly contains vacuolar sap; therefore, it could reflect Cd/Zn transport into the vacuoles indirectly. Up to 12h after exposure to 500nM Cd, there was no difference in the Cd concentration of the root cell sap among two lines (Fig. 4A). However, at 24h after Cd exposure, the Cd concentration in the cell sap was significantly higher in the OX than in the WT (Fig. 4A).

As Zn concentration was much higher in the roots of the *OsHMA3*-overexpressed line (Fig. 2C), the Zn concentration in the root cell sap was also determined. The Zn concentration in the root cell sap was always higher (2.1–4.0 times) in the OX than in the WT (Fig. 4B).

**Fig. 5.**
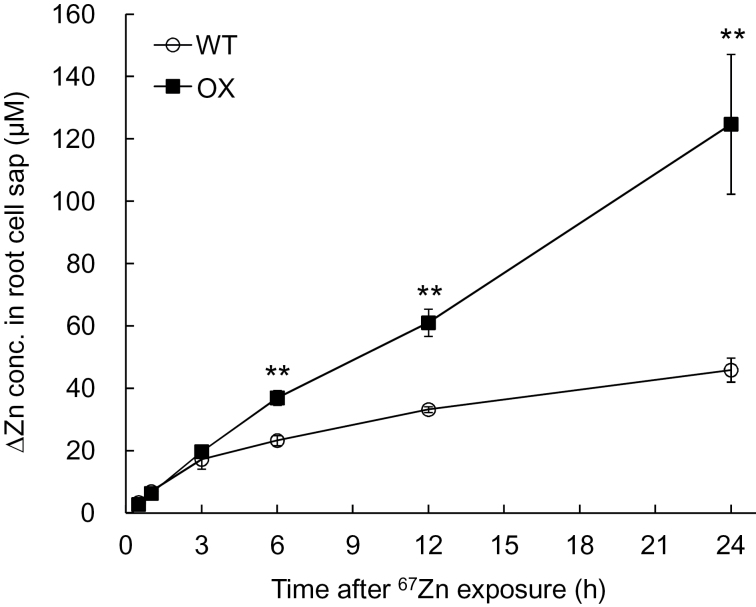
Time-dependent accumulation of ^67^Zn in the roots. Seedlings of an OsHMA3 overexpressed line (OX) and non-transgenic wild-type rice (WT, cv. Nipponbare) were exposed to 500nM ^67^Zn for different times. The concentration of newly accumulated Zn (∆Zn) was calculated by ^66^Zn/^67^Zn ratio determined with isotope mode of ICP-MS. Data are means±SD of four biological replicates. Asterisks indicate significant difference from WT at **P<0.01 by Student’s t-test.

To examine how fast Zn newly taken up is accumulated in the roots, a labelling experiment with ^67^Zn stable isotope was performed. Compared with the WT, the OX showed a higher ^67^Zn accumulation from 6h after the exposure to ^67^Zn (Fig. 5). At 24h, OX accumulated more than three times ^67^Zn more in the roots than the WT.

### Uptake of other divalent metals

**Fig. 6.**
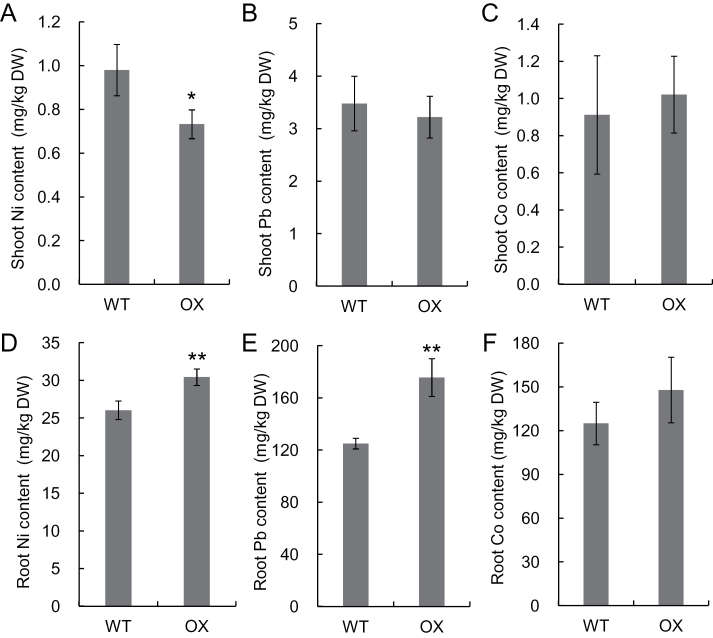
Effect of *OsHMA3* overexpression on uptake of Ni, Pb, and Co. Seedlings of an *OsHMA3* overexpressed line (OX) and non-transgenic wild-type rice (WT, cv. Nipponbare) were exposed to 500nM Ni, Pb, or Co for 24h. The concentration of metals was determined using ICP-MS. Data are means±SD of four biological replicates. Asterisks indicate significant difference from WT at *P<0.05 and **P<0.01 by Student’s t-test.

To further examine whether overexpression of *OsHMA3* also affects the uptake of other divalent metals, we exposed both WT and OX to Pb, Ni, and Co for 24h. The shoot Ni concentration was slightly lower in OX than in WT (Fig. 6A–C), but there was no difference in the concentration of Pb and Co between OX and WT. In the roots, the concentration of Ni and Pb was slightly higher in the OX than in the WT, but no difference in the Co concentration was found between the two lines (Fig. 6D–F).

### Expression of ZIP genes in the *OsHMA3*-overexpressed line

**Fig. 7.**
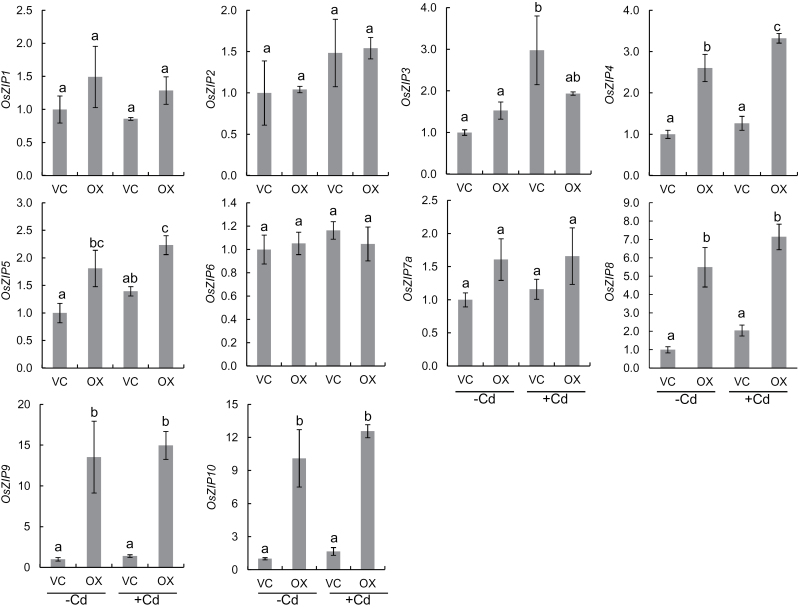
Expression of *ZIP* genes in the roots. Seedlings of an *OsHMA3* overexpressed line (OX) and vector control (VC) were exposed to 0 or 200nM Cd for 24h. The expression of ten *ZIP* genes in the roots was determined by quantitative real-time PCR. *Histone H3* and *Actin* was used as internal standards. Expression relative to VC (–Cd) is shown. Statistical comparison was performed by one-way ANOVA followed by the Tukey’s test. Data are means±SD of three biological replicates. Different letters indicate significant difference at *P*<0.05.

To understand the mechanisms responsible for the increased Zn in the roots, but unchanged Zn in the shoots of OX, we compared the expression level of ten genes belonging to the ZIP family between the vector control and the overexpressed line. Among them, the expression level of *OsZIP4*, *OsZIP5*, *OsZIP8*, *OsZIP9*, and *OsZIP10* was significantly higher in the overexpressed line than in the vector control irrespectively of Cd treatment (Fig. 7), whereas there was no significant difference in other ZIP gene expression.

## Discussion

### Overexpression of *OsHMA3* enhanced the Cd tolerance in rice

*OsHMA3* is mainly expressed in the roots at low level and the expression is not induced by Cd in wild-type rice (Ueno *et al.*, 2010). Overexpression of *OsHMA3* under control of the maize ubiquitine1 promoter resulted in reduced accumulation of Cd in the shoots of brown rice, but did not alter the concentration of iron and zinc (Ueno *et al.*, 2010). In the present study, we found that overexpression of *OsHMA3* also enhanced the tolerance to toxic Cd (Fig. 1). This effect could be attributed to the decreased Cd concentration in the shoots of OX (Fig. 2). Cd displaces essential metals such as Zn, Fe, and Ca from a number of metalloproteins owing to their chemical similarity (Verbruggen *et al.*, 2009). Cd also binds to sulfhydryl residues of constituent proteins or enzymes because of its high affinity for sulfur, resulting in the dysfunction of these proteins. Therefore, high Cd concentration in the shoots causes growth inhibition. When exposed to 1000nM Cd, the shoot Cd concentration in the OX was 20mg kg^–1^, whereas that in the WT was higher than 100mg kg^–1^ (Fig. 2B).

The decreased Cd concentration in the shoots of OX is caused by enhanced sequestration of Cd into the vacuoles in the roots. OsHMA3 is a tonoplast-localized transporter for Cd (Ueno *et al.*, 2010). Loss of function of OsHMA3 results in low accumulation of Cd in the roots, but high accumulation in the shoots (Ueno *et al.*, 2010). By contrast, overexpression of functional *OsHMA3* enhances vacuolar sequestration of Cd in the roots, resulting in accumulation of Cd (Fig. 2B). This enhanced vacuolar sequestration also increases tolerance of the roots to Cd (Fig. 1B).

### Zinc homeostasis is maintained by up-regulating ZIP genes in the *OsHMA3*-overexpressed line

Overexpression of *OsHMA3* did not affect the Zn concentration in the shoots and brown rice (Fig. 2D, Ueno *et al.*, 2010), but increased Zn concentration in the roots (Fig. 2C). Similarly to Cd, the Zn concentration in the root cell sap was always higher in OX than in the WT (Fig. 4). Furthermore, a labelling experiment with ^67^Zn showed that Zn was accumulated in the roots as fast as Cd in the overexpression line (Fig. 5). These results indicate that OsHMA3 is also able to transport Zn into the vacuoles in addition to Cd when it is overexpressed. *OsHMA3* is usually expressed at a low level in natural rice cultivars. Therefore, loss of function of *OsHMA3* did not affect the Zn concentration much in the roots (Ueno *et al.*, 2010; Ueno *et al.*, 2009).

If OsHMA3 is also responsible for sequestration of Zn into the vacuoles in the roots of an *OsHMA3*-overexpressed line, the question arises how the overexpression line is maintained at a similar Zn level to that of WT in the shoots (Fig. 2D). This is different from Cd because overexpression of *OsHMA3* decreased Cd concentration in the shoots although both Cd and Zn were increased in the roots of the overexpression line (Fig. 2A, B). Zn as an essential element is required for many biological processes (Krämer *et al.*, 2007). Therefore the homeostasis of Zn is strictly regulated at different steps including uptake, translocation, and distribution (Olsen and Palmgren, 2014). Many transporters belonging to ZIP (ZRT-IRT-like protein), MTP (metal tolerance protein) and HMA (heavy metal ATPase) families have been proposed to be involved in Zn transport (Olsen and Palmgren, 2014). Expression analysis revealed that five genes (*OsZIP4*, *OsZIP5*, *OsZIP8*, *OsZIP9*, and *OsZIP10*) belonging to the ZIP family were constantly up-regulated in the overexpression line in both the absence and presence of Cd (Fig. 7). Although the exact roles of these ZIP genes are unknown, OsZIP4 and OsZIP8 have been proposed to be involved in Zn uptake/translocation (Ishimaru *et al.*, 2005; Lee *et al.*, 2010a; Bashir *et al.*, 2012) and OsZIP5 was implicated in Zn distribution (Lee *et al.*, 2010b). These findings suggest that translocation of Zn to the shoots is compensated by up-regulating these ZIP genes, which are involved in Zn uptake/translocation/distribution in the *OsHMA3*-overexpressed line.

A homologue of OsHMA3 in *Arabidopsis*, AtHMA3 shows transport activity for Pb and Cd when expressed in a yeast mutant (Gravot *et al.*, 2004). Ectopic overexpression of AtHMA3 improved plant tolerance to Cd, Co, Pb, and Zn (Morel *et al.*, 2009). However, different from OsHMA3, overexpression of AtHMA3 resulted in increased accumulation of Cd in *Arabidopsis* shoot (Morel *et al.* 2009). On the other hand, AhHMA3 identified from a Zn-hyperaccumulating plant, *Arabidopsis halleri*, shows transport activity for Zn (Becher *et al.*, 2004). We found that OsHMA3 is able to transport Cd and Zn in its overexpressed line (Figs 4 and 5), although the transport activity for Zn and Co was not detected when expressed in yeast (Ueno *et al.*, 2010). Overexpression of *OsHMA3* only gave slight effect on uptake of Pb and Co (Fig. 6). The concentration of Ni in the overexpression line of *OsHMA3* was also increased in the roots, but decreased in the shoots (Fig. 6). This trend is similar to that of Cd although more changes was observed for Cd (Fig. 2). There is a possibility that OsHMA3 also transports Ni, although the affinity may be lower compared with Cd. The mechanism underlying the different transport substrate specificity and affinity of HMA3 from different plant species remains to be examined in future.

The concentration of Fe in both the roots and shoots was unaffected by overexpression of *OsHMA3* (Fig. 3). Rice takes up Fe as either ferrous iron or as an Fe–mugineic acid complex (Ishimaru *et al.*, 2006). After it is taken up, Fe may be complexed with ligands, which could not be transported by OsHMA3. By contrast, the concentration of Cu and Mn was somewhat affected by overexpression of *OsHMA3* (Fig. 3A, B, E, F). However, different from Zn, there was no difference in the concentration of Cu and Mn between OX and VC in the absence of Cd (Fig. 3A, B, E, F). Furthermore, unlike Zn, the concentration of Cu and Mn was also increased in the roots of OX. This increase may be caused indirectly by Cd-inhibited growth although this needs to be further examined (Fig. 1).

In conclusion, overexpression of *OsHMA3* enhanced the tolerance to Cd toxicity by increasing sequestration of Cd into vacuoles of root cells and subsequently decreasing translocation of toxic Cd to the shoots. OsHMA3 is also able to transport Zn, but the Zn concentration in the shoots is maintained by up-regulating ZIP genes involved in Zn uptake/translocation/distribution in the *OsHMA3* overexpressed line. Our results indicate that overexpression of *OsHMA3* is an efficient way to reduce Cd accumulation in the grain and to enhance Cd tolerance in rice because unlike knockout of *OsNramp5* and *OsHMA2* there was no negative effect on growth.

## Supplementary data

Supplementary Table S1. Primer sequences for ZIP genes.

Supplementary Data
