# Supplementary material for: Overexpression of OsHMA3 enhances Cd tolerance and expression of Zn transporter genes in rice
Source: J Exp Bot. 2014 Aug 23;65(20):6013–21. doi: 10.1093/jxb/eru340 (PMC4203134; doi:10.1093/jxb/eru340)
Supplement: Supplementary Data [file supp_65_20_6013__index.html]

Overexpression of OsHMA3 enhances Cd tolerance and expression of Zn transporter genes in rice — Overexpression of OsHMA3 enhances Cd tolerance and expression of Zn transporter genes in rice — Supplementary Data 

# Overexpression of *OsHMA3* enhances Cd tolerance and expression of Zn transporter genes in rice

## Supplementary Data

Data files

**Files in this Data Supplement:**

- Supplementary Data - Supplementary Data
