# Supplementary material for: Overexpression of OsHMA3 enhances Cd tolerance and expression of Zn transporter genes in rice
Source: J Exp Bot. 2014 Aug 23;65(20):6013–21. doi: 10.1093/jxb/eru340 (PMC4203134; doi:10.1093/jxb/eru340)
Supplement: Supplementary Data [file supp_eru340_jexbot128223_file001.pdf]

Over-expression of *OsHMA3* enhances Cd tolerance and expression of Zn transporter genes in rice. *Akimasa Sasaki, Naoki Yamaji, and Jian Feng Ma*

Table S1. Primer sequences used for gene expression

Gene	RAP-DB	Forward (5'-3')	Reverse (5'-3')
OsZIP1	Os01g0972200	CGTCATGGCTGTCGTCATGATCTG	AATGGGGTGATAGAAATCGAACATG
OsZIP2	Os03g0411800	TTTCGGACGTTTGTTGGTTC	TCCTGAAACTTTGGTTGGAGT
OsZIP3	Os04g0613000	GCATTGTTCAGGCTAATTTTAAGG	GGCAGTTGAGCTATGCACATTG
OsZIP4	Os08g0207500	TCACTGAGGCCGTCGTCAATCAGG	ACGACAAGTGCGGTCGAGCTGT
OsZIP5	Os05g0472700	CATGAAGACCAAGGTGCAGAGAAGG	TCACGCCCAGATGGCGATCA
OsZIP6	Os05g0164800	TCTACATGGCACTTGTCGATCTC	GACATGGATGCAGATCCAAGCA
OsZIP7a	Os05g0198400	CCTTGCAATCTGGGCCTGAA	CAGATTAGTCTCACGCCCATGA
OsZIP8	Os07g0232800	GGTGCAGAGCAAAGGCAAGCT	AATTTCCTCTACATTAGTCCCTGA
OsZIP9	Os05g0472400	ATCTTCTTCTCGCTAACCACAC	GCAGCCGCTGCGTCGAGAAT
OsZIP10	Os06g0566300	GCTCAGTTAAAGAACTTCTCTGC	CGACATCGAGTCCAGAATTCC
OsActin		GACTCTGGTGATGGTGTCAGC	GGCTGGAAGAGGACCTCAGG
OsHistoneH3		GGTCAACTTGTTGATTCCCCTCT	AACCGCAAAATCCAAAGAACG
